# A Call for Action to Safely Deliver Oral Health Care during and Post COVID-19 Pandemic

**DOI:** 10.3390/ijerph17186704

**Published:** 2020-09-15

**Authors:** Marco Farronato, Santosh K Tadakamadla, Mir Faeq Ali Quadri, Shashidhar Acharya, Jyothi Tadakamadla, Robert M. Love, Mohamed Jamal, Riaan Mulder, Cinzia Maspero, Davide Farronato, Alexander Ivanov, Dirk Neefs, Maria Grazia Cagetti, Danila de Vito, Rishi J. Gupta, Stephen Thaddeus Connelly, Gianluca M. Tartaglia

**Affiliations:** 1 Department of Biomedical, Surgical and Dental Sciences, School of Dentistry, University of Milan, 20122 Milan, Italy; marco.farronato@unimi.it; 2 School of Dentistry and Oral Health, Griffith University, Gold Coast, QLD 4222, Australia; santoshkumar.tadakamadla@griffithuni.edu.au (S.K.T.); jyothi.tadakamadla@griffithuni.edu.au (J.T.); r.love@griffith.edu.au (R.M.L.); 3 Department of Preventive Dental Sciences, College of Dentistry, Jazan University, Jazan 45142, Saudi Arabia; dr.faeq.quadri@gmail.com; 4 Department of Public Health Dentistry, Manipal College of Dental Sciences, Manipal Academy of Higher Education, Manipal 576104, India; sh.acharya@manipal.edu; 5 Hamdan Bin Mohammed College of Dental Medicine, Mohammed Bin Rashid University of Medicine and Health Sciences, Dubai 505055, UAE; mohamed.jamal@mbru.ac.ae; 6 Department of Restorative Dentistry, University of the Western Cape, Cape Town 7535, South Africa; rmulder@uwc.ac.za; 7 Fondazione IRCCS Ca’ Granda, Ospedale Maggiore Policlinico, via Francesco Sforza 35, 20122 Milan, Italy; cinzia.maspero@unimi.it; 8 Department of Medicine and Surgery, School of Dentistry, University of Insubria, 21100 Varese, Italy; davide@farronato.it; 9 Pediatric Cranio-Maxillo-Facial Department—Central Research Institute of Dental and Maxillofacial Surgery, Moscow 119021, Russia; dr.ivanov@cleft.ru; 10 Dierick Dental Care & B-dent Dental Clinic, 2000 Antwerp, Belgium; neefsdirk@gmail.com; 11 WHO Collaborating Centre for Epidemiology and Community Dentistry of Milan, 20142 Milan, Italy; maria.cagetti@unimi.it; 12 Department of Basic Medical Science, Neuroscience and Sense Organs, University of Bari Aldo Moro, 70121 Bari, Italy; danila.devito@uniba.it; 13 San Francisco Veteran’s Affairs Health Care, Department of Oral and Maxillofacial Surgery, University of California San Francisco, San Francisco, CA 94143, USA; rishi.gupta@gmail.com (R.J.G.); stephen.connelly@ucsf.edu (S.T.C.)

**Keywords:** coronavirus, quarantine, professional practice, angiotensin II, pandemics, mouth

## Abstract

The severe acute respiratory syndrome coronavirus 2 (SARS-CoV-2) outbreak started just a couple of months ago and it grew rapidly causing several deaths and morbidities. The mechanism behind the transmission of the virus is still not completely understood despite a multitude of new specific manuscripts being published daily. This article highlights the oral cavity as a possible viral transmission route into the body via the Angiotensin converting enzyme 2 receptor. It also provides guidelines for routine protective measures in the dental office while delivering oral health care.

## 1. Introduction

The recent ongoing outbreak of the SARS-CoV-2 was officially declared a pandemic on the 11th of March 2020 by the World Health Organization [1]. The origin of the infection, causing COVID-19 disease (Coronavirus disease 2019), is related to a mutation at the whole-genome level of a virus belonging to the genus β-Coronavirus, bat-SL-CoVZC45 and bat-SL-CoVZXC2, both normally found in wild non-aquatic mammalians of the order of Chiroptera (Rhinolophus affinis bat) showing 88% identity with a peak in the S2 protein demonstrating around 93% of sequence homology and 98.7% in the E gene [2]. There have been an ever-increasing number of papers published on COVID-19 since January 2020. According to Pubmed, there have been more than fifty thousand papers published [3], at a publication rate of almost 250 per day.

The estimated basic reproduction number (R0) of COVID-19 is around 2.2. It means that on average, each patient spreads infection to 2.2 people [4]. The mechanism behind the transmission of the human–human infection still needs to be understood in more detail. In particular, understanding specific function and location of virus-host interactions are extremely important to prevent further transmission, to protect health care workers (HCW) and to outline the safety guidelines going forward [5]. The norms and operating procedures for HCW who tend to work in close contact with general public—such as dentists, doctors, nurses and other health personnel—will be of extreme importance for the safety of the community because no specific drugs or vaccines are yet available for COVID-19 with sufficient supporting evidence of their efficacy and safety [5]. Early diagnosis and management are still crucial for containing the outbreak.

HCW have been addressed as the most affected by the contagion according to the Italian National Institute of Health [6]. Dentists and other oral health professionals, collectively called Dental Health Care Workers (DHCW), who work in close proximity of the oral cavity could be at even more risk due to the frequent use of instruments which generate aerosols that could be contaminated by the virus [7]. Particularly, with all the non-urgent treatment procedures having been delayed during the lockdown, dental practices are now encountering a swarm of back-logged patients. Therefore, new personal protective equipment (PPE) protocols have to be adopted to effectively prevent the dental offices from becoming a new nidus of viral transmission in the community.

Moreover, during normal daily interactions, contact is more frequently related to functions of the mouth, rather than other sources of transmission such as the nose, the eyes, or the urogenital tract [8]. Angiotensin converting enzyme 2 (ACE2) have been identified as cell entry receptor for SARS-CoV-2 [9,10,11]. The virus attaches to its punitive receptor (ACE2) via binding the S1 region of the spike (S) protein, which must be exposed through an initial enzymatic step mediated by a serine protease transmembrane serine protease 2 (TMPRSS2) or through a co-expressed membrane endopeptidase of the ACE2 receptor, Furin. ACE2/TMPRSS2 has been shown to be expressed in Type 2 alveolar cells in the lung and ACE2/Furin has also been recently demonstrated to be expressed on oral epithelial cells [12]. Thus, there is evidence for viral entry via both the lungs and oral cavity. 

In this paper, we aim to present information from the existing literature about the transmission of SARS-CoV-2, focused on evidence of transmission via the oral cavity. It also aims to gather data to provide evidence-based guidelines which will be of vital importance for determining the protection strategies to adopt while delivering health care, primarily focusing on DHCW’s and their practices.

## 2. Methods

### 2.1. Search for Literature

We initially searched for relevant literature in the National Library of Medicine (MEDLINE via Pubmed) in March 2020, which was repeated again in June 2020. The search was conducted using the keywords “covid-19”, “sars-cov-2”, “dentistry”, “transmission”, “saliva”, and “recommendations”; appropriate Boolean logic was used. We found 38 articles as appropriate. 

### 2.2. Preliminary Clinical Experience 

The guidelines proposed in this review were implemented at SST Dental Clinic in Segrate (MI), Italy during the Italian phase 2 from 15th of May 2020 to 15th of July 2020 under the clinical coordinator supervision.

The ethics committee of the clinic approved the use of serological tests (Ethic committee number IRB022020 doc SO 02) and patients requiring testing were informed about the procedure. After all their questions were answered, they (or their legal representatives in case of minors) agreed to take part in the test by signing the informed consent form. Data includes all the patients attending the clinic for routine dental care who were on the clinic waiting list during the lockdown. All the patients provided signed consent as indicated by the National Dental Association’s guidelines to be self-declared as healthy and fit to work for dental consultation. Data were collected by the clinical process manager using Statistiche Trattamenti grid (Clinical manager Version: 3.1.0.0).

For outcomes identified as missing, clinical notes were reviewed and data were added retrospectively if available. 

All DHCW working in the clinic were tested with serological quick test for IgM/IgG detection on 15 May 2020.

The colloidal gold-based immunochromatographic (ICG) strip targeting viral IgM or IgG antibody was used. Its sensitivity and specificity were previously investigated by Pan et al. [13]. 

## 3. Results

### 3.1. The Oral Cavity

The oral cavity is purported to be one of the main host sites, both for entry and transmission, implicated in SARS-CoV-2 spread either through contact, droplet, aerosols, or saliva. SARS-CoV-2 has been isolated in saliva and recent studies highlight how contamination of the oral environment could occur via fecal-oral transmission for an extended period of time [14,15,16,17]. Even though Bai et al. recommend caution while interpreting their data for the possible influence of confounders, it has been reported that the viral load in potential spreaders’ feces has RNA persistent for nearly five weeks after the patients swab tested negative [18]. 

Based on the above evidence, it might be reasonable to suggest that the risk of transmission through saliva could also last for up to five weeks and this possibility needs significant consideration while making decisions on appropriate precautions in the dental office. Chen et al. [19] confirmed the presence of SARS-CoV-2 in salivary glands in 75% of the critically ill patients. It can be easily drawn that, excluding direct inhalation, the oral cavity could be a critical source of infection among the population. This is further dependent on the size of the particles involved. Droplets and aerosolized particles between 10 and 100 µm tend to deposit in the upper airway and particles under 5–10 µm in size can penetrate deep into the lungs [20]. Thus, for all but the smallest particles, the oral cavity could be one of first sites of viral entry. This could be consistent with the evidence that shows the sense of taste is affected in more than 60% of mildly symptomatic individuals [21]. According to Hamming et al. [22], ACE2 expression was observed in the basal layer of the non-keratinizing squamous epithelium mucosa of the nasal, oral cavity, and nasopharynx, whereas they did not observe its expression on the surface of epithelium. This could suggest that these anatomical areas are not the primary site of entrance for SARS-CoV but areas of virus deposit where favorable environmental conditions for its replication exist. Normal and breathing on exertion result in aerosol production, that would easily reach alveolar pneumocytes in the lower respiratory tract where ACE2 is expressed by endothelial cells from small and large arteries and veins [22]. Human ACE2, responsible for the inhibition of the angiotensin 2, has high-affinity binding capacity to both SARS-CoV and SARS-CoV-2 coronaviruses. There is evidence that considers it as the main co-receptor involved in the infection, but it is suggested that it might not be the only one involved [19,23]. In the initial stages of infection, cholesterol-rich lipid rafts play a fundamental role in viral entry into the cell [24]. These membrane microdomains facilitate both the interaction between the viral spike protein and the cellular ACE2 receptor [25] and the endocytosis process that takes place in the early stages of internalization of coronaviruses [26,27,28].

The importance of cholesterol and other lipids that constitute the lipid rafts is confirmed by a study that shows that cholesterol-supplemented mouse fibroblasts show increased susceptibility to fusion with murine hepatitis virus [29]. In vitro cell models expressing the ACE2 membrane protein have shown that depletion of cholesterol by metil-β-cyclodextrin halves the number of bonds with viral S glycoproteins [25]. Therefore, targeting host lipids via the use of cyclodextrins seems a very promising antiviral strategy [29,30]. Nevertheless, the possibility to interfere with ACE2 receptor glycosylation, which could help to prevent SARS-CoV-2 binding to target cells, should be considered in the therapeutic management of COVID-19 [31].

Moreover, ulcers and periodontal pocket bleeding would mix with the saliva and become part of the particles produced by breathing, exacerbating the risk to the lower respiratory tract for viral penetration [19].

### 3.2. Considerations or Recommendations for Effective Delivery of Oral Health for Dental Hospitals and Clinics

Here we propose a series of suggestions and a clinical protocol that should be taken into consideration by DHCW in hospitals and clinics supplemented by current best evidence and their national guidelines. The proposed protocol was implemented by the lead author (G.M.T.) and colleagues in their daily practice post lockdown. Since its implementation, there is no evidence of cross infection of SARS-CoV-2 between patients and DHCW and vice-versa. 

For this protocol to be implemented efficiently there is a need to understand a few important considerations associated with aerosol generation [20]:

1. The mechanism of spread (contact, droplet, or aerosol);
2. The minimum viral titer and length of exposure required to cause an infection for each of these modes of spread
3. Factors that increase host susceptibility to infection;
4. Host factors that predispose to more severe COVID-19 disease.

Evidence suggests that the classic mechanism of transmission, contact and droplet spread, can be contained mostly by isolating symptomatic patients and by the use of facial masks/facial coverings, which de facto provides a physical barrier to the oral cavity and nose, the primary source of infection for droplets and larger aerosol particles. Thus, patients and all DHCW (clinical and non-clinical) should be educated and encouraged to wear high quality face masks on a regular basis. 

SARS-CoV-2 has been shown to survive in a closed environment for at least 3 h with an estimated half-life of 1 h [32]. Increased airflow and treatment room air exchange rates will be important actions to modify viral particles that remain suspended in micro-aerosols in the environment. If adequate high-volume air exchange mechanics are not in place, then it would be reasonable to rotate use of patient treatment rooms with a 1–3 h quarantine time between use. If complete disinfection of all the surfaces and air change cannot be guaranteed after each patient, the use of disposable coverings should be considered together with disinfection of the surfaces at reach of the contact with the patient’s body. In addition, measures for spatial distancing of >2 m between every non-operating person, including the waiting room, need to be implemented. Careful scheduling of appointments could also help in ensuring spatial distancing by limiting the number of patients scheduled at one point in time. 

The potential appointments should be encouraged to follow the protocol of ‘calling-in, before walking-in’. A structured questionnaire has to be readily available to implement a phone-triage, and all patients entering the dental practice should be treated as potential carriers. The dominant signs and symptoms of COVID-19 that should not be missed during triage are fever, cough, and fatigue, while other rare clinical features that should also be taken into consideration are congestion, rhinorrhea, sore throat, and diarrhea [33]. However, the evidence on the symptoms of COVID-19 is ever changing with new epidemiological research. Dental practices should consider placing placards and warnings in their facility necessitating the use of face masks, avoiding touching of the face, and other recommendations prescribed by the regulatory agencies in their respective countries. These clinical considerations should be considered to be applied compulsorily. If complete disinfection of all the surfaces and air change cannot be guaranteed after each patient for every dental practice (i.e., rapid checks or visits), the use of disposable covering should be considered together with disinfection of the surfaces at reach of the contact with the patient’s body.

The salivary system is activated during the motion of the mandible and with the function of speech, this could represent a source of contagion at the reception and operating room, face masks could help prevent such transmission. In addition, contact with documents, papers, and all the shared objects should be avoided as much as possible [34]. ‘Calling-in, before walking-in’ protocol would help reducing contact with documents and billing should be encouraged while cash payments should be discouraged [35]. Digital documents or mobile applications should be used if required, for instance to sign the consent.

Along with currently known disease confinement measures, access to toilets should be limited in the health care facilities and the frequency of cleaning and disinfection of the toilets needs to be increased. In addition, strict preventive measures should be in place for the eateries and dining areas, given that fecal shedding of SARS-COV-2 could cause significant environmental contamination [36].

Those patients who require emergency treatment but present COVID-19 symptoms should be delayed or referred to another facility with negative pressure room. Unless the practice is equipped with negative pressure treatment rooms, the patients should not be scheduled for dental procedures and referred to such facility. DHCWs should refer to their governmental guidance for treatment and referral of these patients to ensure no ethical and treatment protocols are compromised. In addition, medical advice should be sought in special circumstances, such as patients with COVID-19 symptoms, who are elderly or who have underlying medical comorbidities [37,38,39].

Other measures include use of a pre-procedural mouthwash with active ingredients that decrease the virus load (hydrogen peroxide, povidone iodine, high molecular weight combination of cetylpyridinium chloride and hyaluronic acid) [40,41,42]. The four-handed technique is suggested along with the use of rubber dam complementary to saliva ejectors to reduce the production of aerosols [43].

The use of clothing should include: microparticulated respirators (N95 authenticated by the National Institute for Occupational Safety and Health or FFP2-standard respirators set by the European Union as an equivalent to N95) combined with wearable facial transparent screens and disposable water-resistant shoes, hair and full body covers [44,45].

**Figure 1:**
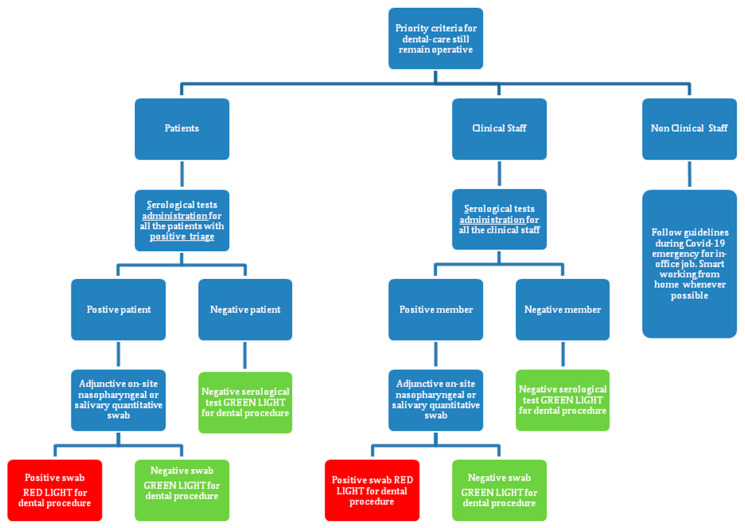
Proposed COVID-19 testing criteria for staff and patients in dental practices during the COVID-19. Positive triage indicates presence of fever and/or cough and/or at least two of the following symptoms: sore throat, headache, diarrhea, vomit, asthenia, muscle pain, joint pain, and loss of taste or smell in the past 15 days prior to the consultation or with PCR blood test positive for IgM/IgG. Red light, dental procedure is recommended to be postponed; Green light, dental procedure can be safely delivered.

Proposed COVID-19 testing criteria and treatment procedures based on COVID-19 diagnosis in dental practice are summarized in Figure 1. In particular, it would be important to have knowledge of the exposure/infection status of clinical staff and patients to SARS-CoV-2. In this regard, it would be pivotal to rely on serological tests for specific anti-SARS-CoV-2 IgM (low affinity) or IgG (high affinity), although there are some concerns of cross-reactivity with phylogenetically related coronaviruses causing common cold remains [46]. Conversely, to diagnose the presence of SARS-CoV-2, nasopharyngeal or salivary swabs followed by viral genome amplification, hopefully not only qualitative but also quantitative, remains currently the most reliable tool [47]. Irrespective of the testing protocol and due to the risk of false negatives, it is of absolute importance that all patients are considered potentially positive. It is expected that DHCW will maintain a high degree of attention adopting the routinely standard infection control precautions.

### 3.3. Preliminary Clinical Experience Results

There were a total of 1278 patients (mean age 47.02 years ± 21.52, minimum 4 years old and maximum 92 years old); 665 females and 613 males, over the 10 weeks period resulting in 2968 clinical consultations. From that, 648 consultations were conducted during the first two weeks post lockdown; 1561 consultations in the entire month of June and 759 consultations during the first two weeks of July. Overall, 671 patients were seen for a single consultation only (e.g., dental hygiene) and 607 patients came for more than one visit (mean 3.72 visits). All the positive triaged patients were investigated with serological quick test for IgM/IgG detection. Twelve positive patients were further investigated with salivary swab but were found to be negative (Table 1).

Three from the total of 32 DHCW were IgG positive. All three of them were detected as negative from the salivary Polymerase Chain Reaction (PCR) swab.

During the period from 15th of May 2020 to 15th of July 2020 one DHCW was isolated due to Covid-19 symptoms, who was tested negative in PCR serological test, therefore returned to work the day after (Table 1).

In total, 15 positive serological tests were observed, which indicates a prevalence of 1.15% (95% confidence interval 0.70–1.88%), in accordance with the prevalence observed in the Italian municipality of city Vo‘ (1.2% (95% CI: 0.8–1.8%) [48].

All consultations were made by appointment and followed the above-mentioned recommendations. The majority of these consultations were for adult service (>16 years old; 89%, *n* = 2642), with 11% (*n* = 326) being for pediatric service and 9.3% (*n* = 246) for orthodontic services.

The first two days of consultations (15–16 May 2020) were used for staff training under the supervision of the clinical coordinator. The number of clinical consultations increased during the first two weeks from 246 in week one to 348 in week two, before stabilizing after week three around an average value of 360 per week corresponding to 65% of the maximum clinical capacity. Both genders were equally represented in those accessing the service (48% male and 52% female). In terms of patient ethnicity, 99.4% of patients attending clinical consultations were recorded as white Caucasian. Black, Asian, and minority ethnic (BAME) made up 0.06%.

Following the above proposed guidelines, no cases COVID-19 disease transmission after single or multiple dental consultations was registered among the DHCW or patients.

## 4. Discussion

Closing down the dental health facilities or limiting the services to emergency treatments for the last few months have undeniably contributed towards slowing down of the rapidly spreading virus. However, further extension of lockdowns or the strategies post-lockdown should be carefully planned after keeping in mind the role played by the DHCW in reducing the burden of oral diseases, which impact people’s general health and quality of life.

Classified as operative and non-operative, depending on their ability to work in the oral cavity or/and provide an essential outside support, the DHCW and the patients visiting the dental practice are undeniably at higher risk of SARS-CoV-2 infection and further transmission [41]. However, careful planning and implementation of strategies based on scientific evidence can be an effective way that may help in adapting to this challenging situation [49,50,51].

The DHCW are bound with the moral obligations to extend their services, but a general consensus is that moral obligations should go hand-in-gloves with evidence-based decision making, and the safety of the health care providers and their patients should be a priority [52]. The current report has presented the readers with protocols concerning the DHCW, which can be regarded as the key steps while marching towards a complete normalcy.

Based on the common experience during this pandemic lockdown from different countries, those patients presenting to the dental emergencies could be classified into four categories already proposed by Izzetti et al. [42]: (1) subjects with known SARS-CoV-2 infection; (2) subjects who have recovered from COVID-19; (3) subjects at potential risk of infection; and (4) subjects with unknown risk of infection, which represents every other patient. It is, however, important that we appropriately identify patients to prevent transmission in the dental practice.

Given that patients could be asymptomatic for prolonged period of time, groups 2 and 3 could be merged into a group of potentially asymptomatic SARS-CoV-2 infected individuals [48]. Moreover, the classification needs to be extended to both clinical and non-clinical staff. Ideally, as health care providers, in time it should be a reasonable expectation that all members of the DHCWs be tested for SARS-CoV-2 and the associated antibody, however, this should not alter the established Covid-19 protective protocols.

With most countries already returning to the new-normal, several recommendations are already in place from their governing bodies.

The main limitation of these recommendations is represented from COVID-19 asymptomatic patients (negative to triage) that are not at the moment correctly identified. The prevalence of asymptomatic cases could only be clarified by a cross-sectional serological survey of the entire population. Similarly, the sample used for presenting these preliminary clinical data consists of mostly Caucasian patients from a single clinic in Italy. The analysis of different samples also including minorities from different countries on large populations is needed.

## 5. Conclusions

The recommendations put forth in this report are based on the currently available scientific information. They have been developed through a consensus gained through discussions between several dental practitioners and researchers across the continents. We do however acknowledge that with rapid developments in the knowledge on transmission of SARS-CoV-2, the recommendations are entitled to change. It is also important to note that different countries are going through different transmission phases, therefore the guidelines proposed here are applicable to those countries which have already returned to new-normal. It is also extremely important that the DHCWs stay abreast with the updated guidelines and recommendations from their respective governments and regulatory agencies. Public fear of dental offices and between DHCWs is still currently high but our experience demonstrates that by following appropriate guidelines and recommendations, cross-infection of SARS-CoV-2 in dental offices could be easily avoided rendering them a safe place for DHCWs and patients too. This is, to our knowledge, the first study that has reported a testing protocol of high exposure volunteers (DHCWs and dental patients). No transmission between the patient and DHCWs in the period of two months was recorded following the proposed guidelines. These data moreover reinforce the importance of epidemic multi-timepoint surveillance of DHCWs and dental patients.

## Figures and Tables

**Table 1:** DHCWs (Dental Health Care Workers) and patients attending dental clinic for consultation stratified for SARS-CoV-2 detection test.

Sample	Number of Patients	Number of Positive Serological Tests for IgM/IgG	Number of Positive Swab Tests
DHCWs			
Pre-symptomatic ^a^	3	3	0
Asymptomatic	29		
Patients			
Pre-symptomatic ^a^	35	12	0
Asymptomatic	1243		
TOTAL	1310	15	0

^a^ Defined as presence of fever and/or cough and/or at least two of the following symptoms: sore throat, headache, diarrhea, vomit, asthenia, muscle pain, joint pain, and loss of taste or smell in the past 1 to 15 days prior to the consultation or with PCR blood test positive for IgM/IgG.
